# Investigation of Intra-fraction Stability and Inter-fraction Consistency of Active Breathing Coordinator (ABC)-Based Deep Inspiration Breath Holds in Left-Sided Breast Cancer

**DOI:** 10.7759/cureus.47047

**Published:** 2023-10-15

**Authors:** Ahamed Badusha Mohamed Yoosuf, Saad Alhadab, Salem Alshehri, Abdulrahman Alhadab, Mamdouh Alqathami

**Affiliations:** 1 Department of Oncology, King Abdulaziz Medical City/Ministry of National Guard Health Affairs, Riyadh, SAU; 2 Clinical Research, King Abdullah International Medical Research Center, Riyadh, SAU; 3 Medicine, King Saud Bin Abdulaziz University for Health Sciences College of Medicine, Riyadh, SAU; 4 Radiological Sciences, King Saud Bin Abdulaziz University, Riyadh, SAU

**Keywords:** image-guided radiotherapy, electronic portal imaging device (epid), left-sided breast cancer, deep-inspiration breath-hold, active breathing coordinator

## Abstract

Background

Deep inspiration breath-hold (DIBH) has been established as a standard technique to reduce cardiac dose. The part of the heart exposed to radiation can be significantly decreased using the DIBH technique during tangential left-sided breast cancer (LSBC) irradiation.

Aim

The objective of this study was to investigate the intra-fraction breath-hold stability and inter-fraction consistency of patient breath-hold against the threshold as a function of air volumes in the setting of active breathing coordinator (ABC)-based DIBH (ABC-DIBH) treatment to LSBC.

Methods

A total of 34 patients treated with external beam radiation therapy (EBRT) to the left breast using the ABC-DIBH device were included. The frequency of breath-holds per fraction and the entire course of treatment along with the total treatment time was evaluated for all patients. A prescription dose of either 200 cGy (conventional) or 267 cGy (hypofractionation) was administered during 649 fractions, resulting in a total of 4,601 breath-hold measurements being evaluated. The amplitude of deviation in terms of air volumes between the baseline threshold and the patient-specific measurement (during each breath-hold) per fraction was used to define the DIBH stability. Likewise, the consistency of the breathing amplitudes was used to define the compliance of patient breath-holds throughout the entire treatment period. Positional accuracy was evaluated using orthogonal (portal) images.

Results

The average number of breath-holds measured over the entire course of treatment for each patient was 144 inspirations (58-351). Similarly, the average number of breath-holds for each fraction during the course of treatment was 11 inspirations (7-21), which included setup imaging and treatment. The total number of breath-holds reduced significantly (p-value < 0.05) with hypofractionation (104 inspirations; range 58-170) as compared to conventional fractionation (145 inspirations; 58-351). The average breath-hold threshold in terms of air volume was 1.41 L (0.6-2.1 L) for all patients. The total treatment time reduced significantly after the third fraction (p-value < 0.05). The average deviation between the measured and baseline threshold breath-holds during the course of treatment was 0.5 L/sec (0.12-1.32 L/sec). The consistency of the breathing amplitudes were maintained within ±0.05 L during the entire treatment for all patients. The average translational shifts measured during setup were 0.28 cm ± 0.3 cm, 0.38 cm ± 0.4 cm, and 0.21 cm ± 0.3 cm in the lateral, longitudinal, and vertical directions, respectively.

Conclusion

The study has demonstrated the variations in intra-fraction breath-hold stability and inter-fraction breath-hold consistency in terms of air volumes for patients who were treated for LSBC. The frequency of breath-holds was observed to be higher with increased total treatment time for the first few fractions and reduced over the course of treatment.

## Introduction

Breast cancer is the most prevalent cancer and the leading cause of mortality among women worldwide. One in four women is diagnosed with breast cancer according to the International Agency for Research on Cancer (IARC) [1]. Breast cancer in Asian females is strongly associated with early onset and a peak incidence rate between 45 and 49 years [2].

Radiation therapy (RT) after breast-conserving surgery in the early stages of breast cancer is the current treatment of care, as RT has proven to improve local control of the tumor and increase overall survival [3]. Most postoperative patients who undergo breast-conserving surgery require whole breast irradiation (WBI) with or without a tumor bed boost, whereas in low-risk patients, a partial breast irradiation (PBI) method can be used. The need for chest wall irradiation (CWI) is less prevalent until it is used in combination with nodal irradiation or post-mastectomy irradiation (PMI) [4].

Involuntary organ movements, such as respiratory motion during RT between and within fractions, may cause dose delivery errors due to excess normal tissue being irradiated and missing target volume [5]. Adverse effects, such as radiation-induced heart disease and cardiovascular events, have been reported in patients with left-sided breast cancer (LSBC) [6-8]. Damage to the heart and lungs due to RT is the major morbid side effect observed after several years of RT treatment [4]. An increase of 1 Gy dose in RT has shown an increased risk of approximately 7% for coronary events; hence, the use of RT should be assessed for the risk-and-benefit ratio for toxicity. In addition, newer methods should be used to prevent such conditions with reduced dose exposure to the heart, lungs, and other organs without the presence of tumors [9].

Strategies currently used to reduce the effects of respiratory motion include integration of respiratory motion into the treatment plan, forced shallow breathing by abdominal compression, deep inspiration breath-holding (DIBH), respiratory gating techniques, and four-dimensional computed tomography (4D CT). Traditionally, DIBH techniques are used, which function by separating the heart from the field of irradiation. DIBH works by inflating the lungs and displacing the heart caudally, which can be used to achieve a lower radiation dose to normal intrathoracic tissues. The DIBH technique enables the reduction of doses to vital organs while maintaining the recommended dose for the tumor [10,11]. Several studies have reported that DIBH RT is a potential method for reducing cardiac doses [12-14].

DIBH reduces the overlap of the heart and lungs with the treatment fields by taking advantage of restricted organ motion during the respiratory cycle [15]. When compared to free breathing, DIBH significantly lowers the dose to the ipsilateral lung for right-sided treatments and the heart for LSBC treatments [11,16]. Daily patient setup and breath-hold reproducibility, which can be checked using cine portal imaging, are crucial for minimizing irradiation of nearby organs at risk (OARs) without sacrificing the treatment of the target volume. The Active Breathing Coordinator (ABC) System (Elekta, Stockholm, Sweden) is one of the commercially available devices developed to reduce radiation exposure to the heart. The system comprises a spirometer and a balloon valve with a mouthpiece set-up that enables the patient to hold their breath and maintain it during the simulation and treatment. The patient's breathing pattern throughout the course of treatment is tracked and recorded by the ABC device. The reproducibility of the patient's lung volume while holding the breath is primarily used to evaluate the effectiveness of the device.

However, DIBH techniques require patient compliance and longer imaging and treatment duration [17]. Throughout the course of therapy, it is necessary to investigate the overall geometric uncertainties, particularly when using various pieces of equipment [18]. Several breath-hold techniques are already available and utilized in normal clinical practice. The instruments used for breath-hold, intra-fraction monitoring, and patient feedback systems differ greatly across the procedures. In general, there is a contrast between voluntary and computer-controlled DIBH approaches, such as surface-guided or spirometry-based systems [15,16,19-21].

The respiration and breath-hold patterns of patients differ during and between fractions. As treatment techniques become more sophisticated through the use of advanced motion management systems, there are uncertainties in the equipment that may impair overall treatment quality. The total geometric uncertainties, especially when more equipment is employed, necessitate investigation during the course of treatment. 

Currently, RT treatment plans for breast cancer are created using two opposing non-divergent isocentric tangent fields along with beam modifiers to homogenize the dose within the target [11,12]. For target-dose homogenization, the tangential field-in-field technique, also known as forward intensity-modulated RT, is employed using a multi-leaf collimator (MLC) [13,14].

The objective of this study was to evaluate intra-fraction stability and inter-fraction consistency of patient breath-holds as a function of air volumes and flow in the setting of ABC-DIBH-based treatment for LSBC. The set-up uncertainty was evaluated using portal images.

## Materials and methods

Patient stratification

In this retrospective study, 34 consecutive patients diagnosed with LSBC who received RT treatment between September 2022 and May 2023 using the ABC-DIBH technique, ABC device (Elekta, Stockholm, Sweden), following breast-conserving therapy or mastectomy, were included. The median age is 46 years old (±10).

Simulation using the ABC-DIBH device

The ABC system uses a spirometer, which consists of a balloon valve, turbine impeller, and optoelectronic sensor to gauge patients' respiratory volumes. The patient's airflow is stopped by the balloon valve at a predetermined breath-hold volume. For a known air volume, the turbine impeller makes one revolution. The opto-electronic sensor tracks rotations and picks up airflow signals.

A GE Discovery CT590 RT CT scanner (GE Medical Systems, Chicago, IL, USA) was used for the simulation using institutional imaging protocols. The patients were simulated in a supine position on a breast board with arms abducted and externally rotated for DIBH using a slice thickness of 5 mm and covered from the mandible to the thorax, encompassing all normal structures. The DIBH technique was used in patients who were able to hold their breath for 20-30 seconds, at least three times, and was performed using an ABC device (Elekta, Stockholm, Sweden). Patients received coaching until they could consistently perform DIBH. Subsequently, a breath-hold was used to obtain a CT image in order to enhance the gap between the heart, chest wall, and breast tissue by taking advantage of lung hyperinflation.

The target and OAR contouring were delineated by a consultant oncologist based on the Radiation Therapy Oncology Group (RTOG) guidelines to define the clinical target volume (CTV) and planning target volume (PTV), which included the entire contralateral chest wall and lymph node region around the collar bone. The OARs included the ipsilateral lung, contralateral lung, contralateral breast, heart, and spinal cord [22,23].

RT treatment planning

Treatment plans were generated using the CMS Monaco (v5.2.11 Elekta, Crawley, UK) treatment planning system (TPS), which utilizes the collapsed cone algorithm. All cases were planned using the three-dimensional conformal therapy (3D CRT) technique. For each patient, the PTV and various OARs were considered. The goal of the treatment plan was to have 95% of the PTV covered by at least 90% of the prescribed dose. OAR dose limits were optimized to be as low as possible according to the quantitative analysis of normal tissue effects (QUANTEC) guidelines and RTOG protocols [23-25]. Either 5000 cGy in 25 fractions (n=17) or 4256 cGy in 16 fractions (n=17) was used. A bolus of 0.5 cm was used in every other fraction for the chest wall cases, and skin involvement was as per the institutional protocols in the respective mentioned fractions.

Image guidance and treatment delivery

Elekta Infinity® (Elekta, Crawley, UK) with an Agility MLC® head was used to deliver all plans. The beam modulator head assembly consisted of 80 leaf pairs for a total of 160 leaves, which were projected at an isocenter width of 5 mm. For all plans, an MV setup image was acquired and registered with digitally reconstructed radiographs to verify the patient setup. The chest wall was used as the landmark for the breast, lung, and heart, whereas the clavicle was utilized as the landmark for lymph nodes. The treatment couch corrections in our institution's DIBH image guidance protocol are based on portal imaging, which includes the acquisition of orthogonal anterior-posterior and lateral MV images at the first three fractions and weekly thereafter. Daily acquisitions were limited to tangential pre-port images (treatment fields) to verify the breath-hold utilizing the distance between the heart and chest wall. For patients with affected lymph nodes, an anterior image is acquired once a week to correct for potential patient rotation. All images were captured while maintaining the pre-set breath-hold setting using the ABC-DIBH device. The delivery of the treatment and ABC device is controlled manually by the radiation therapist. The treatment is paused manually on observing a discrepancy of ±0.05 L in the breath-hold measurement (air volumes) from the threshold level, and the patients would be communicated to re-adjust the breath-hold. Couch corrections were applied if the position errors of the bony structures in the tangential breast images were greater than 4 mm and if the residual errors of the bony landmarks for the lymph node areas were greater than 4 mm and the reference distance between the heart and chest wall deviated by more than 4 mm. To confirm the accuracy of the corrections, more anterior and lateral orthogonal images were obtained at the following two fractions [26]. 

Data extraction using the ABC-DIBH device

During CT simulation and treatment delivery, including daily imaging for setup, the ABC-DIBH device was used to record the air volume of every patient. In this study, the breath-hold volume that the ABC recorded during the CT scan was referred to as the reference breath-hold volume. Every 20 ms, the output data file records the patient's breathing during imaging and treatment. The actual breath-hold volumes held by the patients during treatment were compared to the reference breath-hold volume and analyzed as a function of the airflow volume.

Intra-fraction stability and inter-fraction reproducibility

The frequency of breath-holds per fraction and the entire course of treatment was evaluated for all patients. The breath-hold deviation between the baseline threshold and the patient-specific measurement in terms of air volumes (during each breath-hold) per fraction was used to define ABC-DIBH intra-fraction stability. Similarly, the measurement of air volumes for the entire treatment period (comparing breath-holds of all treatment fractions) was evaluated for the patient-specific inter-fraction ABC-DIBH reproducibility. The consistency of the breathing amplitudes was used to define reproducibility between breath-holds in this context. The difference in air volumes between the minimum and maximum values was calculated and used as a measure of reproducibility. This was done by calculating the breathing amplitudes of all the breath-holds performed on a patient [27,28].

## Results

The demographics, prescription doses, clinical characteristics, staging, and dosimetric parameters of the patients assessed in this study are reported in Table 1. The average monitor unit (MU) was 611.5 MU (±147.8 MU) and the average treatment time for each fraction was 25.7 mins (±1.5 min). All treatment plans generated in this study met the institutional constraints and objectives and were clinically reviewed and approved by consultant radiation oncologists before treatment. Similarly, all cardiac dose indices obtained using all planning techniques met the limits recommended in the literature.

**Table 1:** Patient demographics, tumor characteristics, and dosimetric parameters of the selected cohort of patients ECOG: Eastern Cooperative Oncology Group; AJCC: American Joint Committee on Cancer; T: tumor; N: node; Tis: tumor in situ; n: number; ER: estrogen receptor; PR: progesterone receptor; HER2: human epidermal growth factor receptor

Patient characteristics	Total number of patients N = 31
Median age	46 years (±10)
ECOG	1 (n = 34)
T stage	T1 (n = 7)
	T2 (n = 15)
	T3 (n = 12)
N stage	N0 (n = 19)
	N1 (n = 13)
	Na1 (n = 2)
M stage	M0 (n= 34)
AJCC stage	0 (n = 0)
	I-A (n = 4)
	II-A (n = 9)
	II - B (n = 15)
	III - A (n = 6)
ER	Negative (n = 10)
	Positive (n = 24)
PR	Negative (n = 12)
	Positive (n = 22)
HER-2/neu	Negative (n= 27)
	Positive (n = 7)
Dosimetric parameters	
Prescription dose	5000 cGy / 25# (n = 17)
	4256 cGy / 16# (n = 17)
Total number of breath-holds (range)	147.9 (58 – 351)
Average breath-hold/fraction (range)	6.8 (4 – 12.5)
Threshold	1.4 L (0.6 – 2.1)
Translational shift	Lateral = 0.28 cm (± 0.3 cm)
	Longitudinal = 0.38 cm (± 0.4 cm)
	Vertical = 0.21 cm (± 0.3 cm)

**Figure 1:**
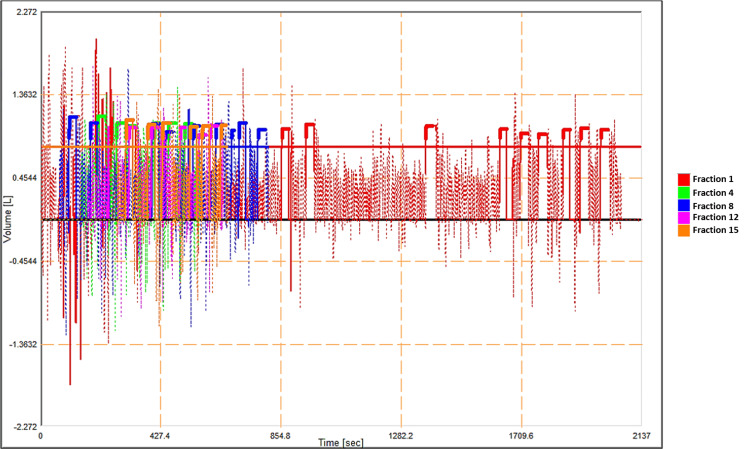
Intra breath-hold stability and inter-fractional consistency of a patient breath-hold during the course of radiotherapy treatment (every fourth fraction). Each color represents the breath-hold patterns during every fourth fraction. Screen capture of ABC-DIBH plots from five different fractions showing the vertical deviation in breath-hold of a patient in volume (L) with respect to the reference threshold value (0.9 L) over the course of treatment.

Figure 1 illustrates the frequency of breath-hold pattern of a patient in terms of air volumes (L) and consistency of breath-hold threshold compliance by the patient over the course of treatment. The breath-hold details from every fourth fraction have been presented with time (sec) over the volume of air (L). As shown in Figure 1, the frequency of inspirations in terms of air volumes that the patient has to perform breath-hold was observed to be higher for the first fraction (red), and the total time recorded for treatment, including setup images, was 36 minutes. The consistencies of the threshold were maintained within ±0.5 L from the reference value, as shown in Figure 1. The treatment or imaging was interrupted manually by observing a discrepancy in the threshold. The frequency of the breath-hold reduced after the third fraction and resulted in treatment times lesser than 14 minutes.

**Figure 2:**
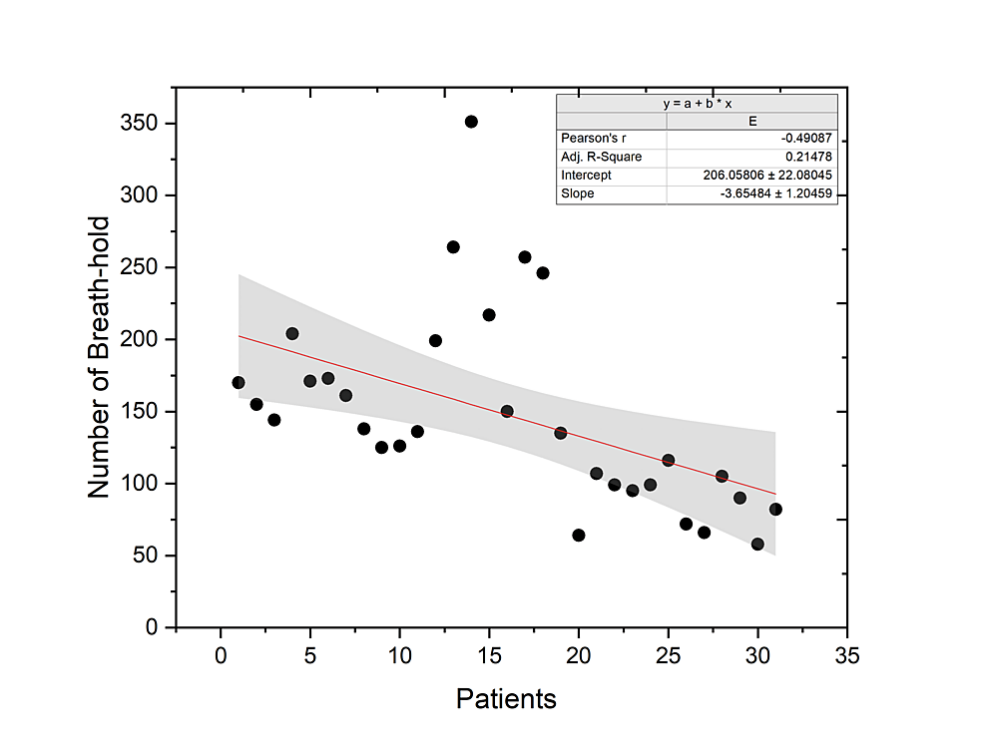
Illustration of total number of breath-holds by each patient during the treatment. The data have been represented as the number of breath-holds (N) for each patient over the course of treatment. The red line is the linear fit plot of the number of breath-holds (with upper and lower 95% confidence bands).

A fractional prescription dose of either 200 cGy or 267 cGy was administered during 649 fractions, resulting in a total of 4,601 breath-holds being evaluated. The average frequency of breath-holds over the entire course of treatment for each patient was measured to be 144 inspirations (15-351), as shown in Figure 2. The average number of breath-holds per fraction by a patient during the course of treatment was 11 inspirations (7-21), which included setup imaging and treatment. The total number of breath-hold inspirations that a patient has to comply reduced significantly (p-value < 0.05) with hypofractionation (104 inspirations; range 58-170) as compared to conventional fractionation (145 inspirations; range 58-351), mainly due to reduced fractionation.

**Figure 3:**
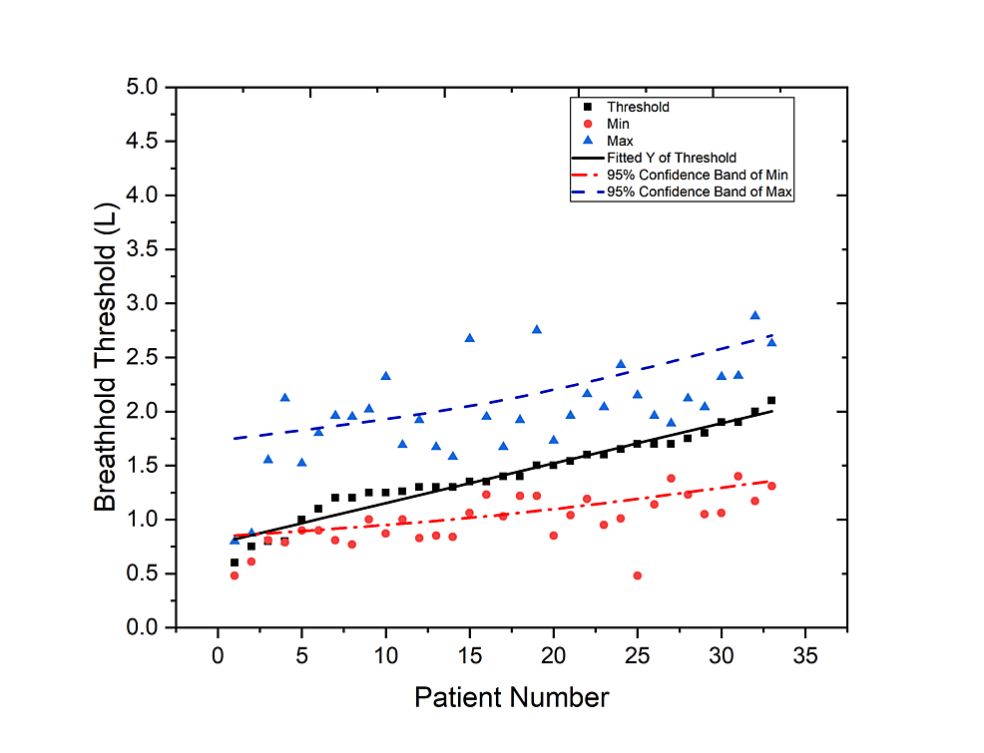
Illustration of breath-hold consistency against the reference threshold value during CT simulation with the minimum and maximum differences from the threshold. The data have been represented with the average, minimum, and maximum breath-holds (L) of each patient. The black line is the linear fit of the breath-hold, whereas the red and blue dotted lines are the 95% confidence band of the minimum and maximum breath-holds.

The ABC device recorded the air volumes as a function of airflow rate throughout the entire radiation treatment. During breath-holding, each patient showed a range of airflow rates. As a result, the differences in the airflow rate during breath-holds were what led to the fluctuations in air volume. Figure 3 illustrates the changes in breath-hold measurements, in terms of the minimum and maximum with respect to threshold values, during the course of treatment for each patient against the threshold measurements. The average breath-hold threshold was found to be 1.41 L/s (0.6-2.1 L/s) for all patients. The average absolute difference in the amount of airflow measured during the course of treatment that are lesser than the breath-hold threshold compliance was 0.43 L (0.0-1.2 L/s). Similarly, the average absolute difference in the amount of air-flow difference more than the breath-hold threshold compliance was 0.57 L/s (0.1-1.3 L/s). The treatment was interrupted if the measured breath-hold was different to the threshold and the patient would be communicated to adjust the breath-hold for further treatment. The average translational shifts measured during setup were 0.28 cm ± 0.3 cm, 0.38 cm ± 0.4 cm, and 0.21 cm ± 0.3 cm in the lateral, longitudinal, and vertical directions, respectively.

## Discussion

The present study addresses the intra-fraction stability and inter-fraction reproducibility of patient breath-holds during the delivery of ABC-based RT treatment to left-sided breast tumors. This study reveals that changes in breath-hold volume, as determined by spirometry, have an impact on doses to the heart over the course of RT. At a given point of time during treatment, if the measured breath-hold of a patient is lesser than the reference breath-hold, it will lead to increased dose to the heart. In terms of breathing characteristics, such as airflow rate, breath-hold time, and threshold, the study found that each patient displayed individual differences. Patient-specific breath-hold settings are essential to increase the accuracy of the OAR dose because reproducibility when using breath-hold platforms for RT is still a concern. Variations in air volume were unavoidable since we set the threshold lower, at around 75% of the maximal breath-hold as recommended by the manufacturer, despite the fact that a consistent breath-hold volume is expected throughout the entire treatment using the ABC breath-hold approach.

In addition, we hypothesize that proper coaching is essential to create a calm environment for treatment as patients' discomfort can result in rapid breathing or complicated breath-holds. Longer treatment sessions may cause patient discomfort after repeated breath-holds; as a result, appropriate coaching is needed to maintain the stable breath-hold comfortably.

The patient compliance to reference breath-hold in terms of air volume increased over the course of treatment. One of the potential explanations was that as the treatment progressed, patients became accustomed to and at ease with the ABC-DIBH. Furthermore, the ability of the patients to maintain a constant lung volume may have increased as a result of improvements in their ability to breathe during the treatment.

Xiao et al. examined over 7,200 DIBH cycles using the surface imaging system AlignRT (VisionRT, London, UK). The median of the 5th to 95th percentile range of translational displacement during a single breath-hold or overall breath-holds throughout the course of a single treatment session was investigated [27]. The current study used only the portal images for verification. Similarly, Reitz et al. investigated the internal accuracy of LSBC treatments using optically monitored DIBH setup with daily image-guided radiotherapy (IGRT) [28,29]. The study reported a maximum absolute setup error of 16.3 mm. The current study analyzed the intra-fraction stability and inter-fraction reproducibility using the patient's breath-hold compliance in terms of air volumes over the course of treatment. In a recent study, Ertan et al. analyzed the reproducibility and variability of the voluntary breath-hold level using a real-time position management device and reported a significant difference in breath-hold level between planning and treatment delivery [30]. 

Moreover, DIBH requires patient adherence to actively breathe in a predefined threshold volume before every treatment field delivery. The average time spent on breath-hold practice before CT simulation was reported to be 20 minutes [31]. Furthermore, inherent limitations of the technology itself need to be considered, such as an increase in the CT simulation time (estimated 45-55 minutes) including coaching and an average increase in the daily treatment time of 25 minutes per patient, which may increase the clinical workload.

Limitations

Continuous portal imaging for position monitoring during treatment delivery has many benefits, but the technique is limited to conformal fields and necessitates the use of X-ray visible markers, such as the chest wall. The analysis of 3D motion is challenging due to the 2D nature of portal images. The DIBH technique used in this study was limited by manual controlling of the ABC device by the radiation therapist during simulation and treatment.

## Conclusions

The study has demonstrated the variations in intra-fraction breath-hold stability and inter-fraction breath-hold consistency in terms of air volumes for patients who were treated for LSBC using the ABC-DIBH device. The average absolute difference in the amount of airflow measured during the course of treatment was maintained within 0.05 L. The frequency of breath-holds was observed to be higher with the increased total treatment time for the first few fractions and reduced after the fourth fraction.
